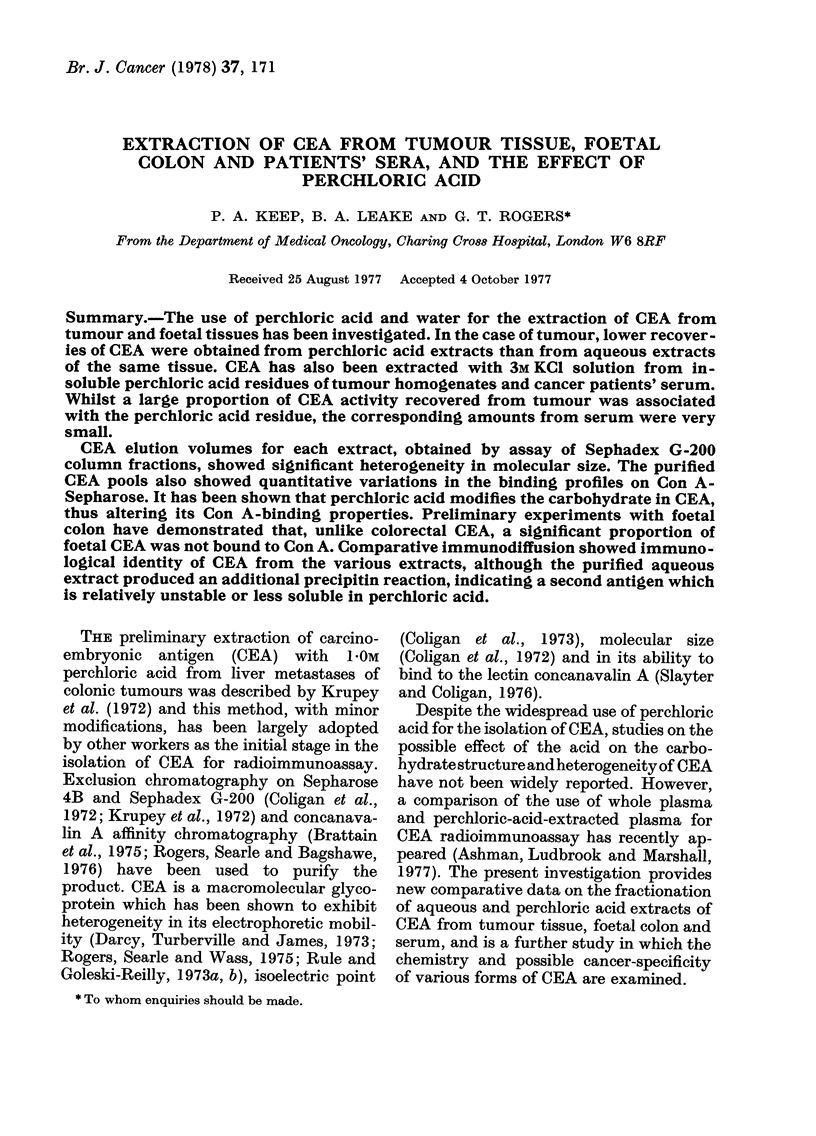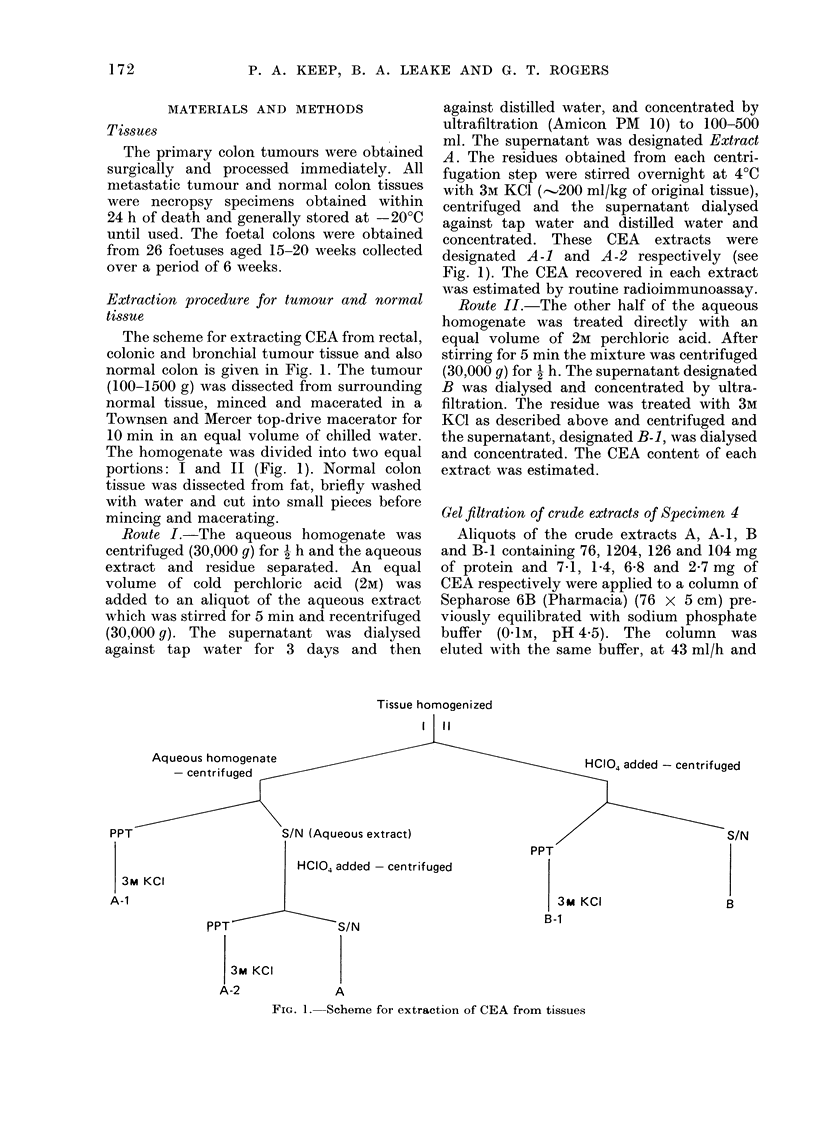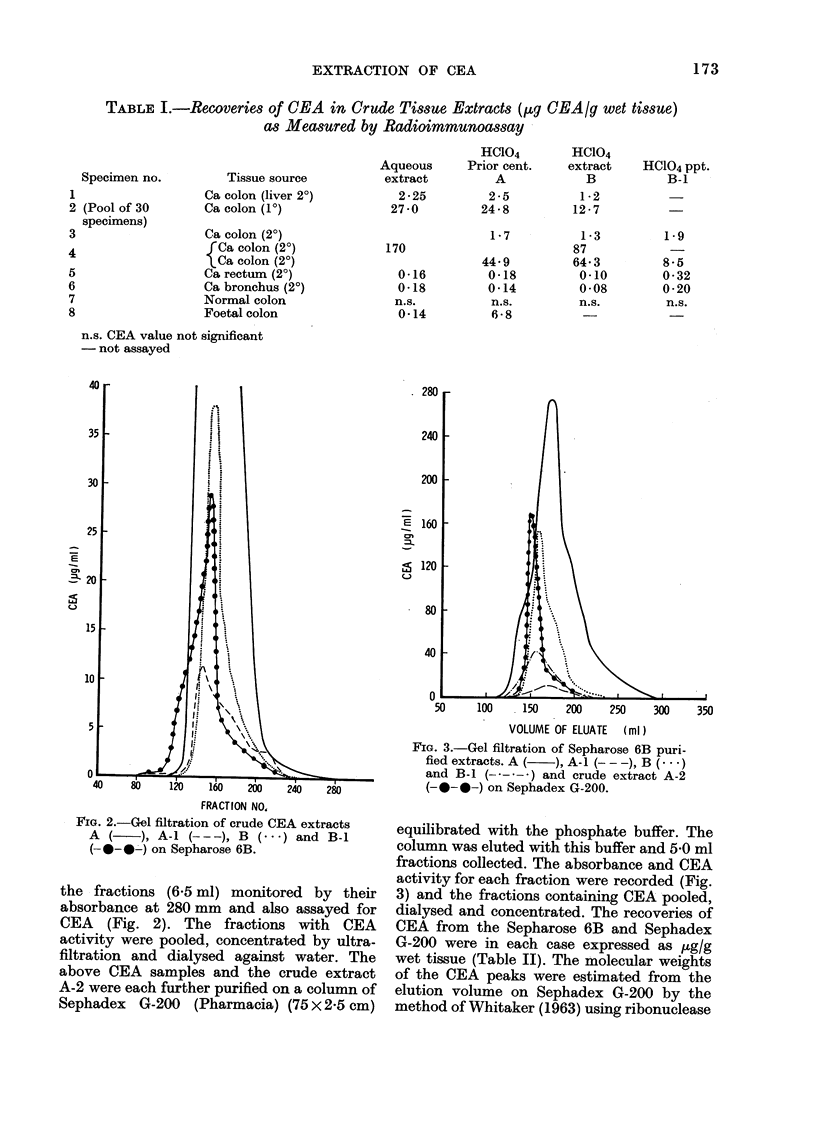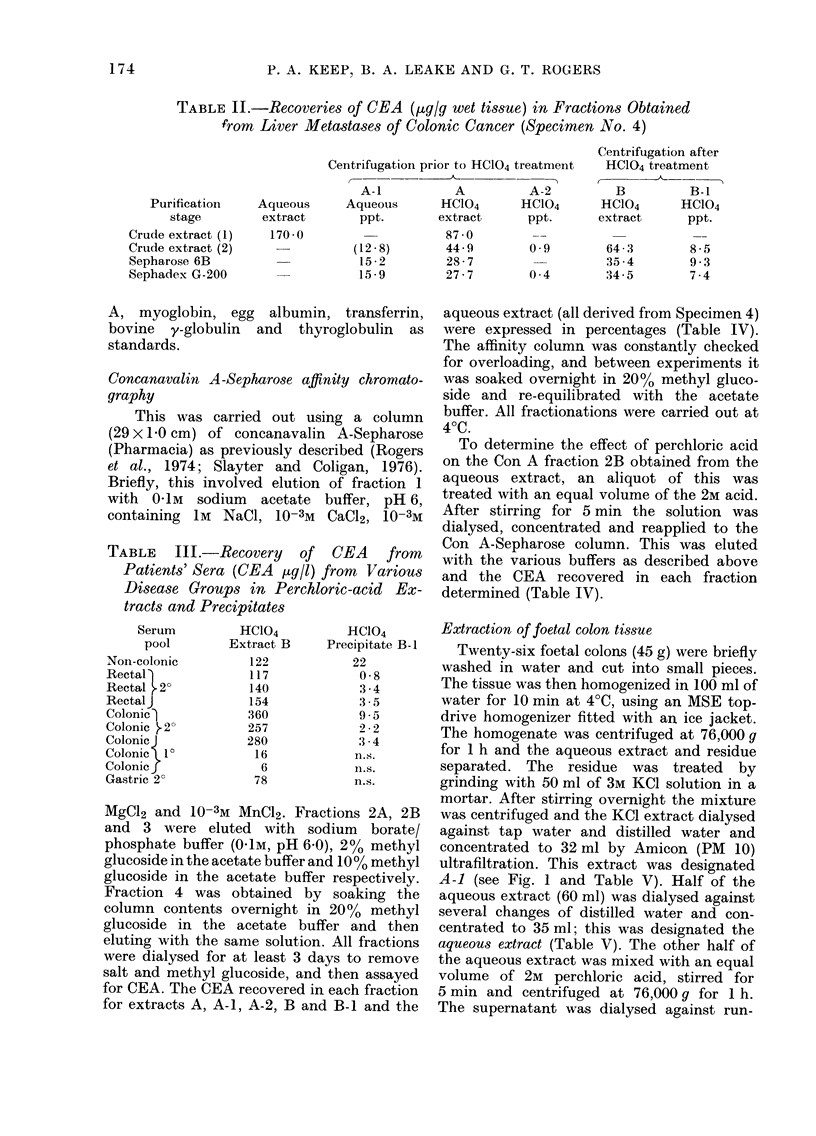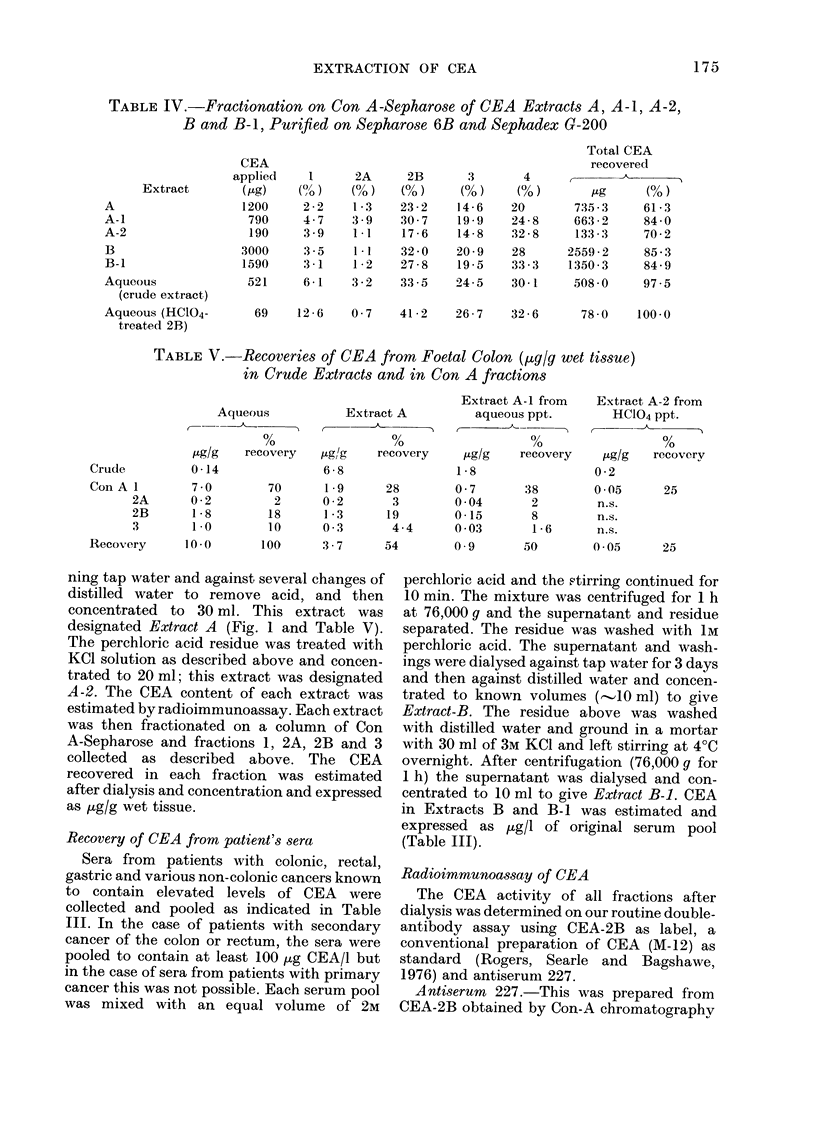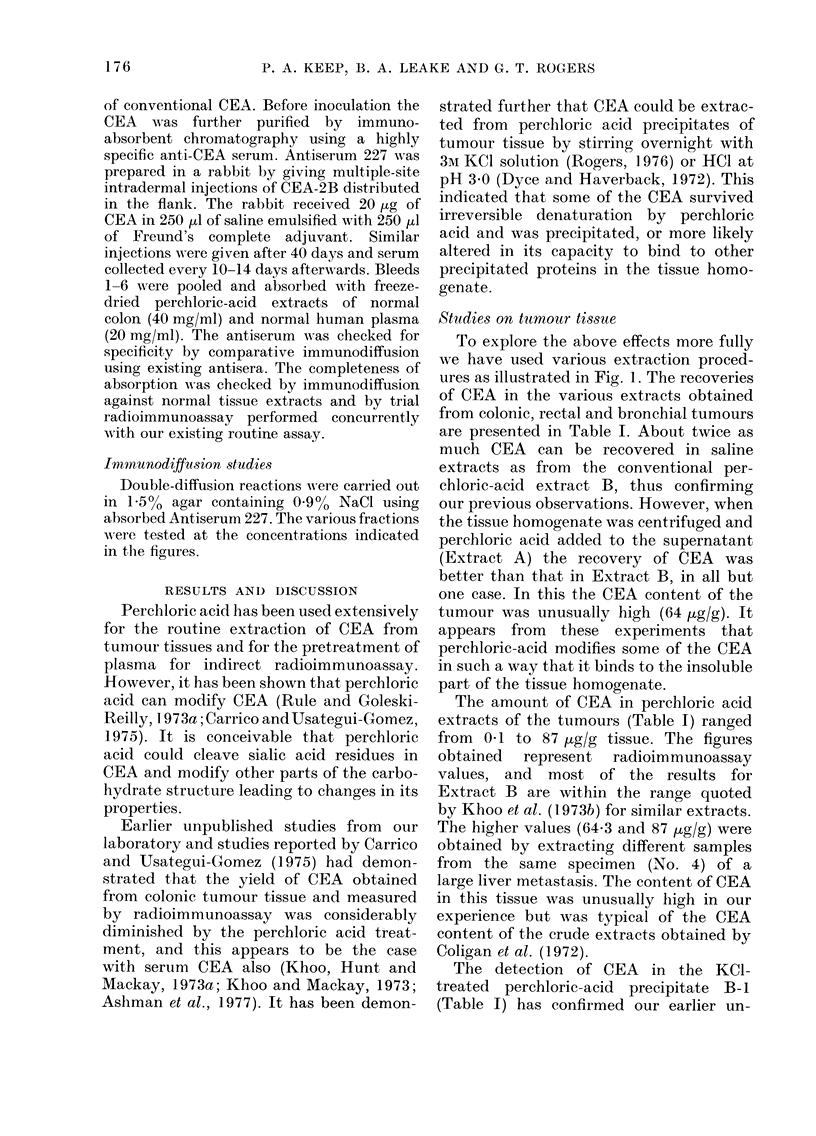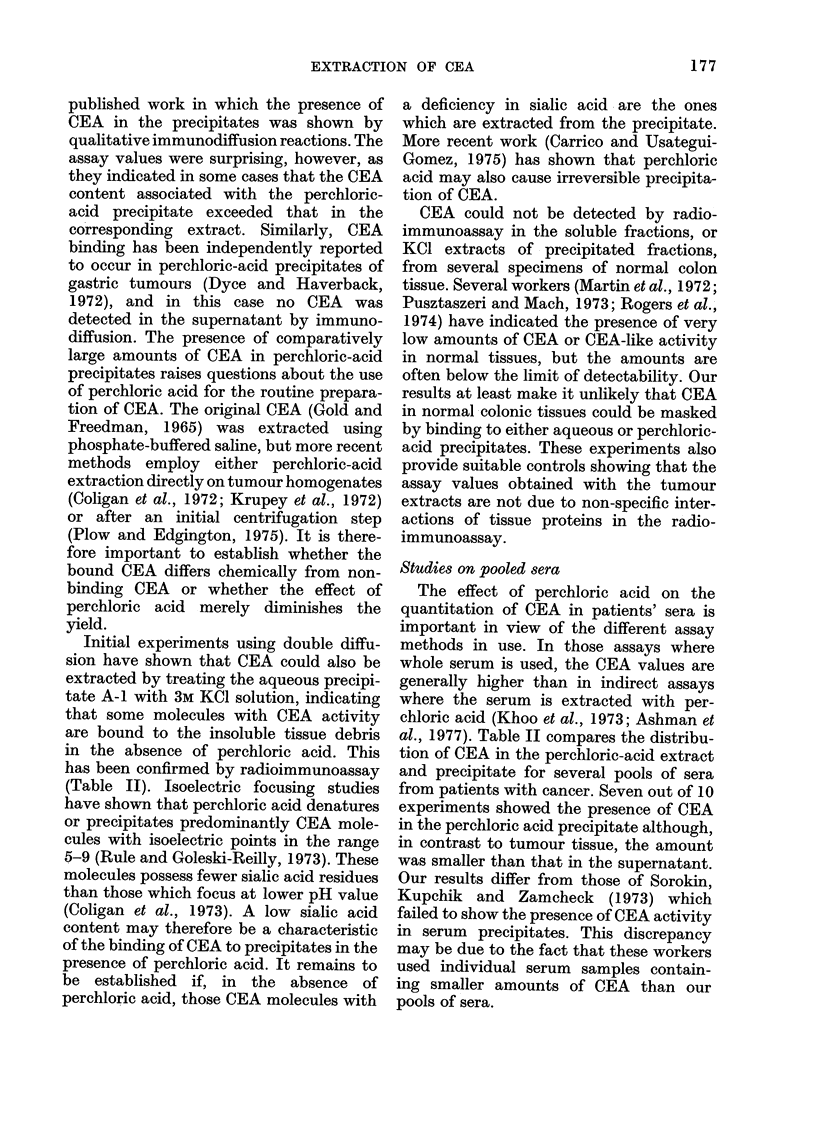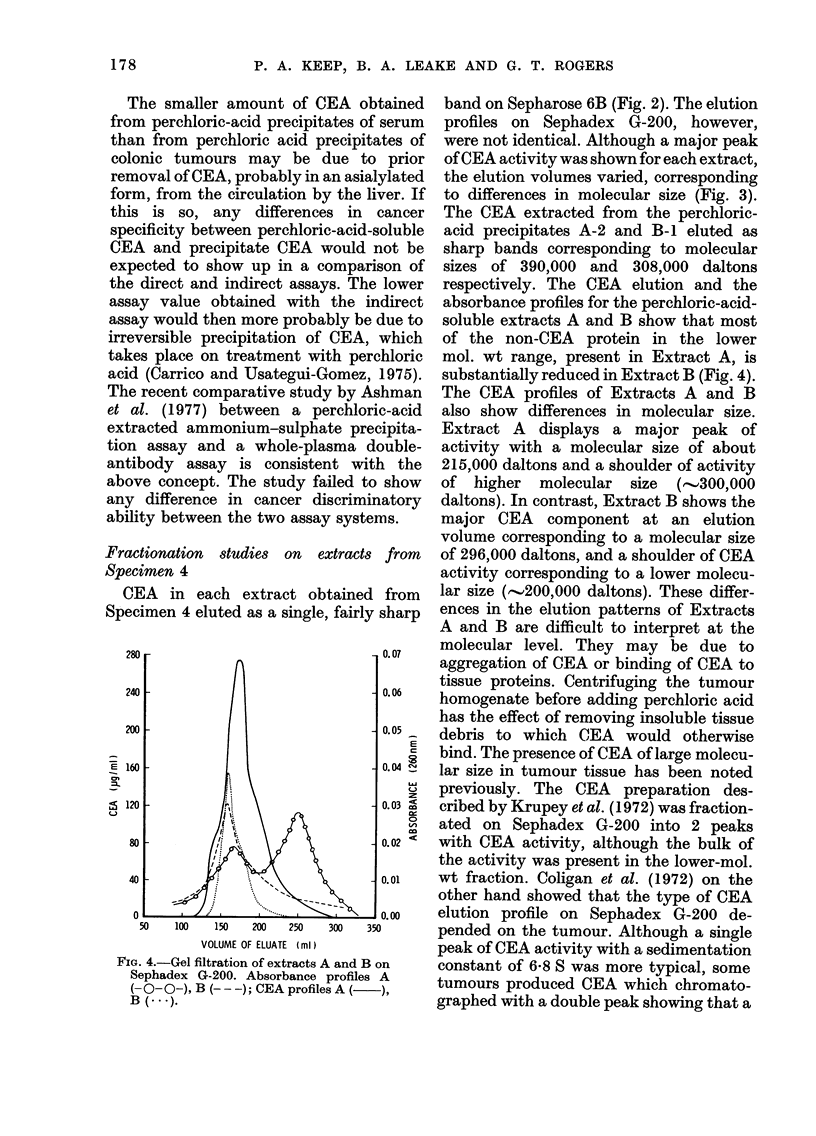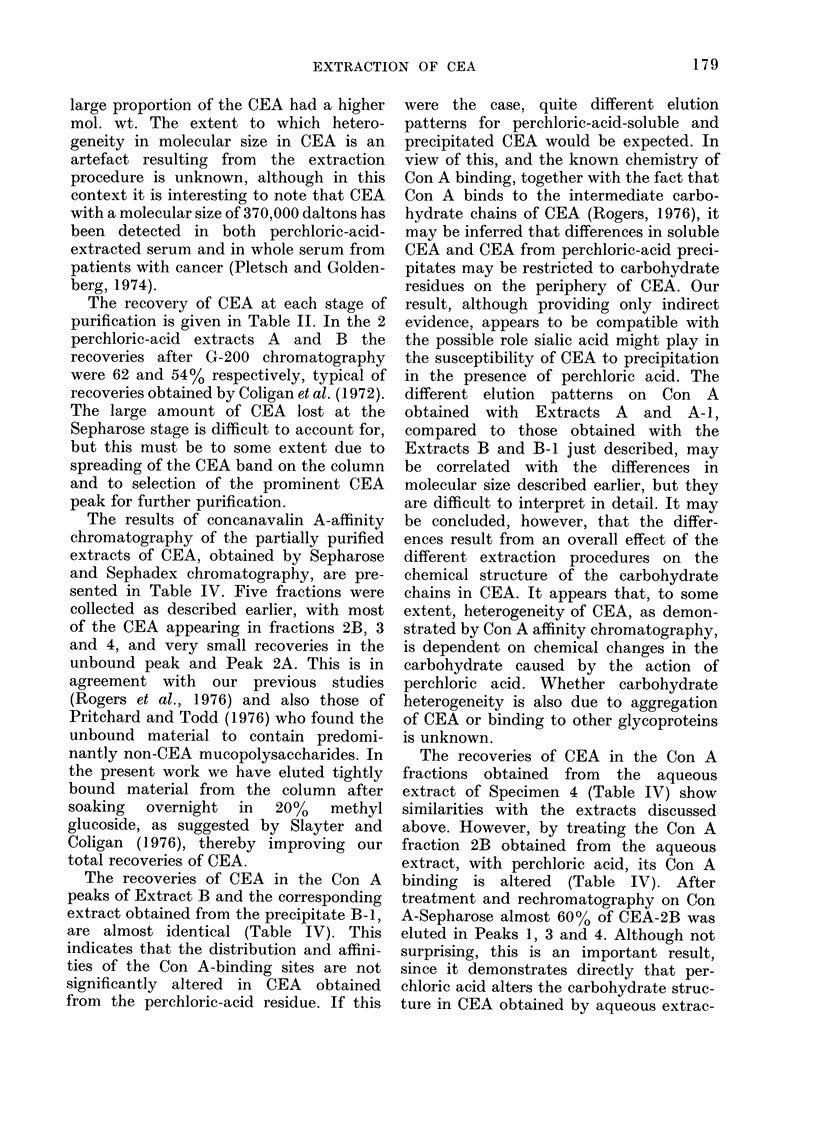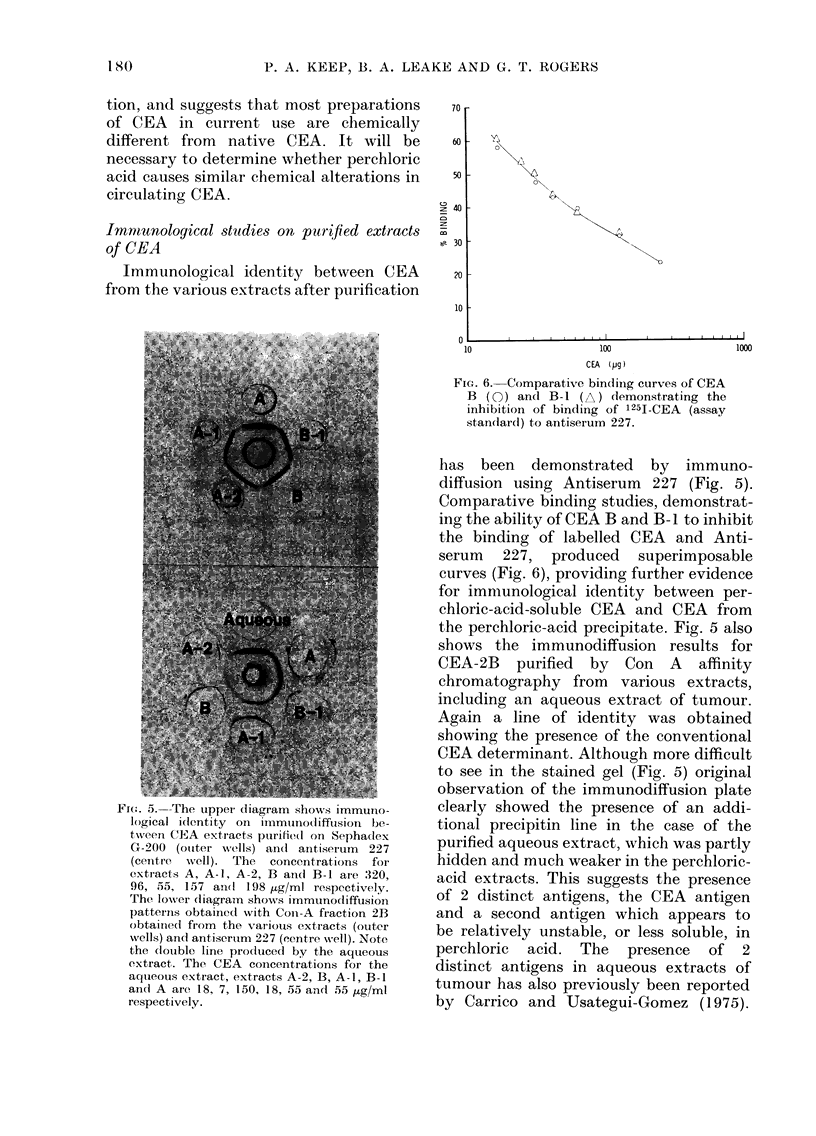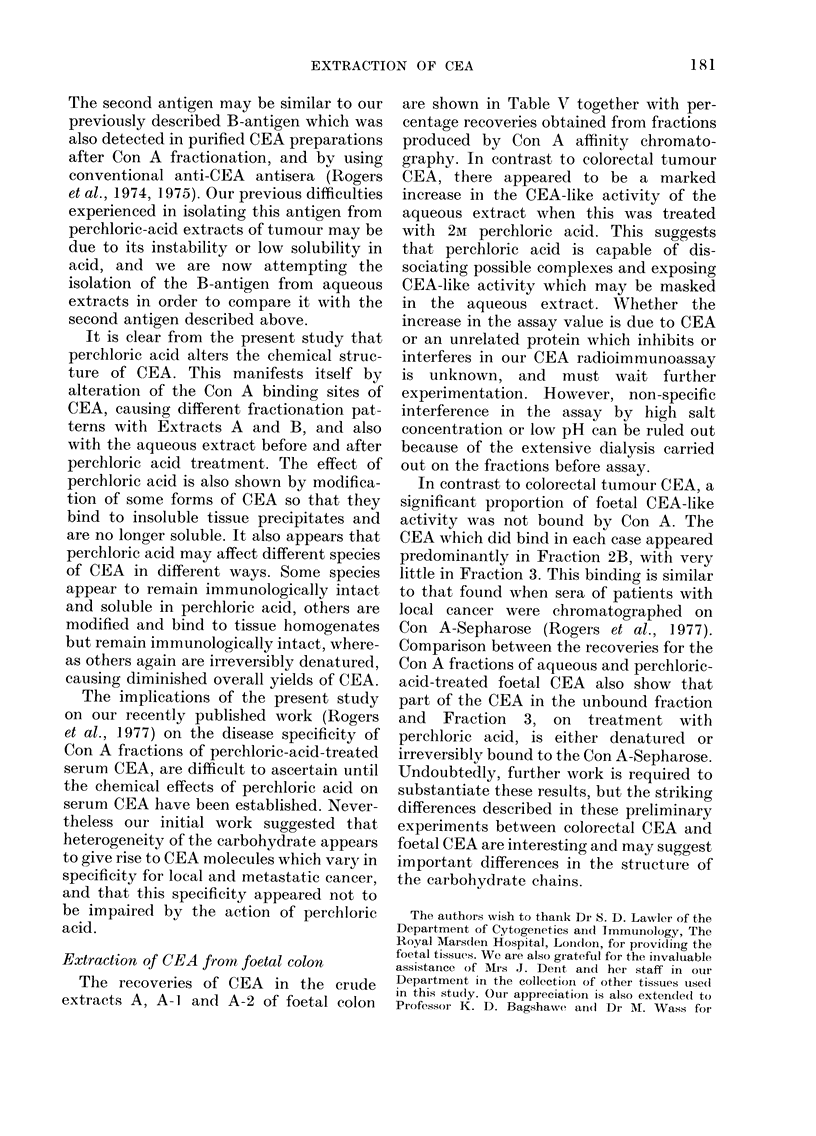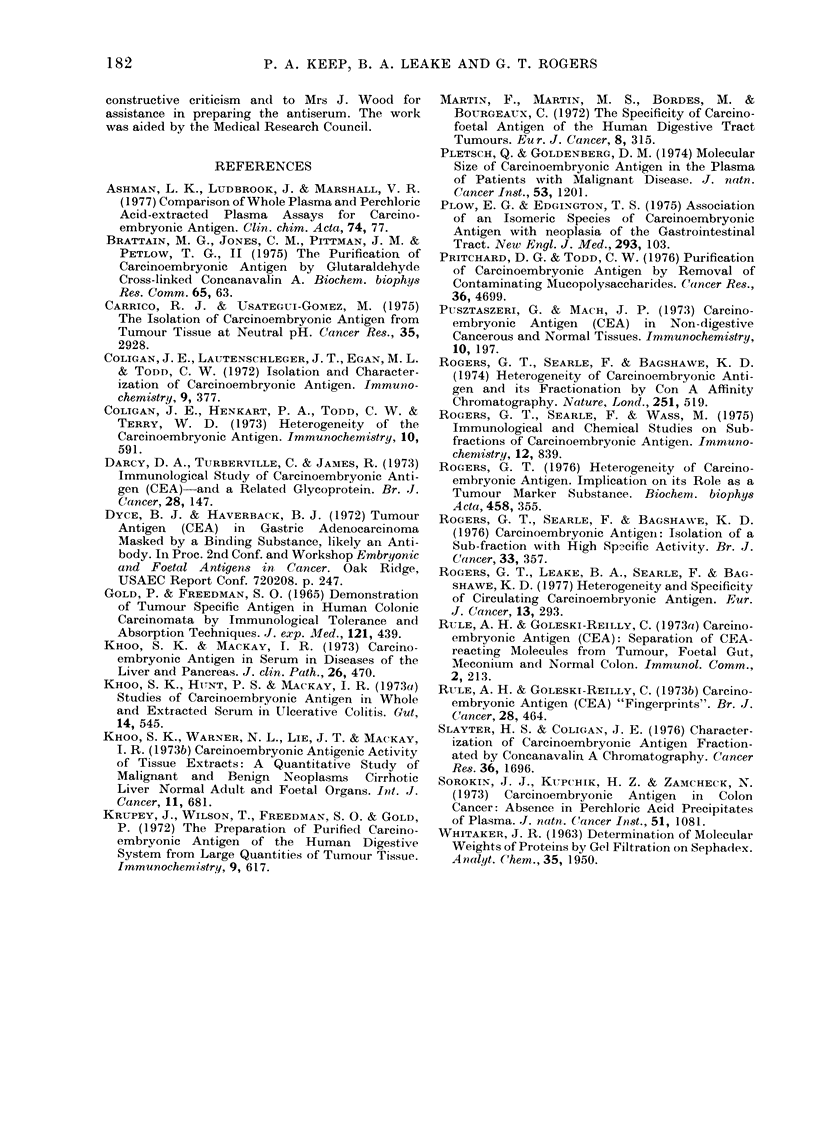# Extraction of CEA from tumour tissue, foetal colon and patients' sera, and the effect of perchloric acid.

**DOI:** 10.1038/bjc.1978.25

**Published:** 1978-02

**Authors:** P. A. Keep, B. A. Leake, G. T. Rogers

## Abstract

**Images:**


*Br. J. Cancer* (1978) **37**, 171

### EXTRACTION OF CEA FROM TUMOUR TISSUE, FOETAL COLON AND PATIENTS' SERA, AND THE EFFECT OF PERCHLORIC ACID

P. A. KEEP, B. A. LEAKE AND G. T. ROGERS*

*From the Department of Medical Oncology, Charing Cross Hospital, London W6 8RF*

Received 25 August 1977 Accepted 4 October 1977

**Summary.—The use of perchloric acid and water for the extraction of CEA from tumour and foetal tissues has been investigated. In the case of tumour, lower recoveries of CEA were obtained from perchloric acid extracts than from aqueous extracts of the same tissue. CEA has also been extracted with 3M KCl solution from insoluble perchloric acid residues of tumour homogenates and cancer patients' serum. Whilst a large proportion of CEA activity recovered from tumour was associated with the perchloric acid residue, the corresponding amounts from serum were very small.**

**CEA elution volumes for each extract, obtained by assay of Sephadex G-200 column fractions, showed significant heterogeneity in molecular size. The purified CEA pools also showed quantitative variations in the binding profiles on Con A-Sepharose. It has been shown that perchloric acid modifies the carbohydrate in CEA, thus altering its Con A-binding properties. Preliminary experiments with foetal colon have demonstrated that, unlike colorectal CEA, a significant proportion of foetal CEA was not bound to Con A. Comparative immunodiffusion showed immunological identity of CEA from the various extracts, although the purified aqueous extract produced an additional precipitin reaction, indicating a second antigen which is relatively unstable or less soluble in perchloric acid.**

THE preliminary extraction of carcinoembryonic antigen (CEA) with 1·0M perchloric acid from liver metastases of colonic tumours was described by Krupey *et al.* (1972) and this method, with minor modifications, has been largely adopted by other workers as the initial stage in the isolation of CEA for radioimmunoassay. Exclusion chromatography on Sepharose 4B and Sephadex G-200 (Coligan *et al.*, 1972; Krupey *et al.*, 1972) and concanavalin A affinity chromatography (Brattain *et al.*, 1975; Rogers, Searle and Bagshawe, 1976) have been used to purify the product. CEA is a macromolecular glycoprotein which has been shown to exhibit heterogeneity in its electrophoretic mobility (Darcy, Turberville and James, 1973; Rogers, Searle and Wass, 1975; Rule and Goleski-Reilly, 1973*a*, *b*), isoelectric point (Coligan *et al.*, 1973), molecular size (Coligan *et al.*, 1972) and in its ability to bind to the lectin concanavalin A (Slayter and Coligan, 1976).

Despite the widespread use of perchloric acid for the isolation of CEA, studies on the possible effect of the acid on the carbohydrate structure and heterogeneity of CEA have not been widely reported. However, a comparison of the use of whole plasma and perchloric-acid-extracted plasma for CEA radioimmunoassay has recently appeared (Ashman, Ludbrook and Marshall, 1977). The present investigation provides new comparative data on the fractionation of aqueous and perchloric acid extracts of CEA from tumour tissue, foetal colon and serum, and is a further study in which the chemistry and possible cancer-specificity of various forms of CEA are examined.

* To whom enquiries should be made.

### MATERIALS AND METHODS

*Tissues*

The primary colon tumours were obtained surgically and processed immediately. All metastatic tumour and normal colon tissues were necropsy specimens obtained within 24 h of death and generally stored at −20°C until used. The foetal colons were obtained from 26 foetuses aged 15–20 weeks collected over a period of 6 weeks.

*Extraction procedure for tumour and normal tissue*

The scheme for extracting CEA from rectal, colonic and bronchial tumour tissue and also normal colon is given in Fig. 1. The tumour (100–1500 g) was dissected from surrounding normal tissue, minced and macerated in a Townsen and Mercer top-drive macerator for 10 min in an equal volume of chilled water. The homogenate was divided into two equal portions: I and II (Fig. 1). Normal colon tissue was dissected from fat, briefly washed with water and cut into small pieces before mincing and macerating.

*Route I.*—The aqueous homogenate was centrifuged (30,000 *g*) for ½ h and the aqueous extract and residue separated. An equal volume of cold perchloric acid (2M) was added to an aliquot of the aqueous extract which was stirred for 5 min and recentrifuged (30,000 *g*). The supernatant was dialysed against tap water for 3 days and then against distilled water, and concentrated by ultrafiltration (Amicon PM 10) to 100–500 ml. The supernatant was designated *Extract A*. The residues obtained from each centrifugation step were stirred overnight at 4°C with 3M KCl (~200 ml/kg of original tissue), centrifuged and the supernatant dialysed against tap water and distilled water and concentrated. These CEA extracts were designated *A-1* and *A-2* respectively (see Fig. 1). The CEA recovered in each extract was estimated by routine radioimmunoassay.

*Route II.*—The other half of the aqueous homogenate was treated directly with an equal volume of 2M perchloric acid. After stirring for 5 min the mixture was centrifuged (30,000 *g*) for ½ h. The supernatant designated *B* was dialysed and concentrated by ultrafiltration. The residue was treated with 3M KCl as described above and centrifuged and the supernatant, designated *B-1*, was dialysed and concentrated. The CEA content of each extract was estimated.

*Gel filtration of crude extracts of Specimen 4*

Aliquots of the crude extracts A, A-1, B and B-1 containing 76, 1204, 126 and 104 mg of protein and 7·1, 1·4, 6·8 and 2·7 mg of CEA respectively were applied to a column of Sepharose 6B (Pharmacia) (76 × 5 cm) previously equilibrated with sodium phosphate buffer (0·1M, pH 4·5). The column was eluted with the same buffer, at 43 ml/h and

FIG. 1.—Scheme for extraction of CEA from tissues

TABLE I.—*Recoveries of CEA in Crude Tissue Extracts (μg CEA/g wet tissue) as Measured by Radioimmunoassay*

| Specimen no. | Tissue source | Aqueous extract | $HClO_4$ Prior cent. A | $HClO_4$ extract B | $HClO_4$ ppt. B-1 |
|---|---|---|---|---|---|
| 1 | Ca colon (liver 2°) | 2·25 | 2·5 | 1·2 | — |
| 2 (Pool of 30 specimens) | Ca colon (1°) | 27·0 | 24·8 | 12·7 | — |
| 3 | Ca colon (2°) | | 1·7 | 1·3 | 1·9 |
| 4 | Ca colon (2°) | 170 | | 87 | — |
| | Ca colon (2°) | | 44·9 | 64·3 | 8·5 |
| 5 | Ca rectum (2°) | 0·16 | 0·18 | 0·10 | 0·32 |
| 6 | Ca bronchus (2°) | 0·18 | 0·14 | 0·08 | 0·20 |
| 7 | Normal colon | n.s. | n.s. | n.s. | n.s. |
| 8 | Foetal colon | 0·14 | 6·8 | — | — |

n.s. CEA value not significant
— not assayed

FIG. 2.—Gel filtration of crude CEA extracts A (——), A-1 (– – –), B (· · ·) and B-1 (–●–●–) on Sepharose 6B.

FIG. 3.—Gel filtration of Sepharose 6B purified extracts. A (——), A-1 (– – –), B (· · ·) and B-1 (–·–·–·) and crude extract A-2 (–●–●–) on Sephadex G-200.

the fractions (6·5 ml) monitored by their absorbance at 280 mm and also assayed for CEA (Fig. 2). The fractions with CEA activity were pooled, concentrated by ultrafiltration and dialysed against water. The above CEA samples and the crude extract A-2 were each further purified on a column of Sephadex G-200 (Pharmacia) (75×2·5 cm) equilibrated with the phosphate buffer. The column was eluted with this buffer and 5·0 ml fractions collected. The absorbance and CEA activity for each fraction were recorded (Fig. 3) and the fractions containing CEA pooled, dialysed and concentrated. The recoveries of CEA from the Sepharose 6B and Sephadex G-200 were in each case expressed as μg/g wet tissue (Table II). The molecular weights of the CEA peaks were estimated from the elution volume on Sephadex G-200 by the method of Whitaker (1963) using ribonuclease

TABLE II.—*Recoveries of CEA (μg/g wet tissue) in Fractions Obtained from Liver Metastases of Colonic Cancer (Specimen No. 4)*

| | | Centrifugation prior to $HClO_4$ treatment | | | Centrifugation after $HClO_4$ treatment | |
|---|---|---|---|---|---|---|
| Purification stage | Aqueous extract | A-1 Aqueous ppt. | A $HClO_4$ extract | A-2 $HClO_4$ ppt. | B $HClO_4$ extract | B-1 $HClO_4$ ppt. |
| Crude extract (1) | 170·0 | — | 87·0 | — | — | — |
| Crude extract (2) | — | (12·8) | 44·9 | 0·9 | 64·3 | 8·5 |
| Sepharose 6B | — | 15·2 | 28·7 | — | 35·4 | 9·3 |
| Sephadex G-200 | — | 15·9 | 27·7 | 0·4 | 34·5 | 7·4 |

A, myoglobin, egg albumin, transferrin, bovine γ-globulin and thyroglobulin as standards.

*Concanavalin A-Sepharose affinity chromatography*

This was carried out using a column (29 × 1·0 cm) of concanavalin A-Sepharose (Pharmacia) as previously described (Rogers *et al.*, 1974; Slayter and Coligan, 1976). Briefly, this involved elution of fraction 1 with 0·1M sodium acetate buffer, pH 6, containing 1M NaCl, $10^{-3}$M $CaCl_2$, $10^{-3}$M $MgCl_2$ and $10^{-3}$M $MnCl_2$. Fractions 2A, 2B and 3 were eluted with sodium borate/phosphate buffer (0·1M, pH 6·0), 2% methyl glucoside in the acetate buffer and 10% methyl glucoside in the acetate buffer respectively. Fraction 4 was obtained by soaking the column contents overnight in 20% methyl glucoside in the acetate buffer and then eluting with the same solution. All fractions were dialysed for at least 3 days to remove salt and methyl glucoside, and then assayed for CEA. The CEA recovered in each fraction for extracts A, A-1, A-2, B and B-1 and the aqueous extract (all derived from Specimen 4) were expressed in percentages (Table IV). The affinity column was constantly checked for overloading, and between experiments it was soaked overnight in 20% methyl glucoside and re-equilibrated with the acetate buffer. All fractionations were carried out at 4°C.

TABLE III.—*Recovery of CEA from Patients' Sera (CEA μg/l) from Various Disease Groups in Perchloric-acid Extracts and Precipitates*

| Serum pool | $HClO_4$ Extract B | $HClO_4$ Precipitate B-1 |
|---|---|---|
| Non-colonic | 122 | 22 |
| Rectal } 2° | 117 | 0·8 |
| Rectal } 2° | 140 | 3·4 |
| Rectal } 2° | 154 | 3·5 |
| Colonic } 2° | 360 | 9·5 |
| Colonic } 2° | 257 | 2·2 |
| Colonic } 2° | 280 | 3·4 |
| Colonic } 1° | 16 | n.s. |
| Colonic } 1° | 6 | n.s. |
| Gastric 2° | 78 | n.s. |

To determine the effect of perchloric acid on the Con A fraction 2B obtained from the aqueous extract, an aliquot of this was treated with an equal volume of the 2M acid. After stirring for 5 min the solution was dialysed, concentrated and reapplied to the Con A-Sepharose column. This was eluted with the various buffers as described above and the CEA recovered in each fraction determined (Table IV).

*Extraction of foetal colon tissue*

Twenty-six foetal colons (45 g) were briefly washed in water and cut into small pieces. The tissue was then homogenized in 100 ml of water for 10 min at 4°C, using an MSE top-drive homogenizer fitted with an ice jacket. The homogenate was centrifuged at 76,000 *g* for 1 h and the aqueous extract and residue separated. The residue was treated by grinding with 50 ml of 3M KCl solution in a mortar. After stirring overnight the mixture was centrifuged and the KCl extract dialysed against tap water and distilled water and concentrated to 32 ml by Amicon (PM 10) ultrafiltration. This extract was designated *A-1* (see Fig. 1 and Table V). Half of the aqueous extract (60 ml) was dialysed against several changes of distilled water and concentrated to 35 ml; this was designated the *aqueous extract* (Table V). The other half of the aqueous extract was mixed with an equal volume of 2M perchloric acid, stirred for 5 min and centrifuged at 76,000 *g* for 1 h. The supernatant was dialysed against run-

TABLE IV.—*Fractionation on Con A-Sepharose of CEA Extracts A, A-1, A-2, B and B-1, Purified on Sepharose 6B and Sephadex G-200*

| Extract | CEA applied (μg) | 1 (%) | 2A (%) | 2B (%) | 3 (%) | 4 (%) | Total CEA recovered μg | (%) |
|---|---|---|---|---|---|---|---|---|
| A | 1200 | 2·2 | 1·3 | 23·2 | 14·6 | 20 | 735·3 | 61·3 |
| A-1 | 790 | 4·7 | 3·9 | 30·7 | 19·9 | 24·8 | 663·2 | 84·0 |
| A-2 | 190 | 3·9 | 1·1 | 17·6 | 14·8 | 32·8 | 133·3 | 70·2 |
| B | 3000 | 3·5 | 1·1 | 32·0 | 20·9 | 28 | 2559·2 | 85·3 |
| B-1 | 1590 | 3·1 | 1·2 | 27·8 | 19·5 | 33·3 | 1350·3 | 84·9 |
| Aqueous (crude extract) | 521 | 6·1 | 3·2 | 33·5 | 24·5 | 30·1 | 508·0 | 97·5 |
| Aqueous ($HClO_4$-treated 2B) | 69 | 12·6 | 0·7 | 41·2 | 26·7 | 32·6 | 78·0 | 100·0 |

TABLE V.—*Recoveries of CEA from Foetal Colon (μg/g wet tissue) in Crude Extracts and in Con A fractions*

| | Aqueous | | Extract A | | Extract A-1 from aqueous ppt. | | Extract A-2 from $HClO_4$ ppt. | |
|---|---|---|---|---|---|---|---|---|
| | μg/g | % recovery | μg/g | % recovery | μg/g | % recovery | μg/g | % recovery |
| Crude | 0·14 | | 6·8 | | 1·8 | | 0·2 | |
| Con A 1 | 7·0 | 70 | 1·9 | 28 | 0·7 | 38 | 0·05 | 25 |
| 2A | 0·2 | 2 | 0·2 | 3 | 0·04 | 2 | n.s. | |
| 2B | 1·8 | 18 | 1·3 | 19 | 0·15 | 8 | n.s. | |
| 3 | 1·0 | 10 | 0·3 | 4·4 | 0·03 | 1·6 | n.s. | |
| Recovery | 10·0 | 100 | 3·7 | 54 | 0·9 | 50 | 0·05 | 25 |

ning tap water and against several changes of distilled water to remove acid, and then concentrated to 30 ml. This extract was designated *Extract A* (Fig. 1 and Table V). The perchloric acid residue was treated with KCl solution as described above and concentrated to 20 ml; this extract was designated *A-2*. The CEA content of each extract was estimated by radioimmunoassay. Each extract was then fractionated on a column of Con A-Sepharose and fractions 1, 2A, 2B and 3 collected as described above. The CEA recovered in each fraction was estimated after dialysis and concentration and expressed as μg/g wet tissue.

*Recovery of CEA from patient's sera*

Sera from patients with colonic, rectal, gastric and various non-colonic cancers known to contain elevated levels of CEA were collected and pooled as indicated in Table III. In the case of patients with secondary cancer of the colon or rectum, the sera were pooled to contain at least 100 μg CEA/l but in the case of sera from patients with primary cancer this was not possible. Each serum pool was mixed with an equal volume of 2M perchloric acid and the stirring continued for 10 min. The mixture was centrifuged for 1 h at 76,000 *g* and the supernatant and residue separated. The residue was washed with 1M perchloric acid. The supernatant and washings were dialysed against tap water for 3 days and then against distilled water and concentrated to known volumes (~10 ml) to give *Extract-B*. The residue above was washed with distilled water and ground in a mortar with 30 ml of 3M KCl and left stirring at 4°C overnight. After centrifugation (76,000 *g* for 1 h) the supernatant was dialysed and concentrated to 10 ml to give *Extract B-1*. CEA in Extracts B and B-1 was estimated and expressed as μg/l of original serum pool (Table III).

*Radioimmunoassay of CEA*

The CEA activity of all fractions after dialysis was determined on our routine double-antibody assay using CEA-2B as label, a conventional preparation of CEA (M-12) as standard (Rogers, Searle and Bagshawe, 1976) and antiserum 227.

*Antiserum 227.*—This was prepared from CEA-2B obtained by Con-A chromatography of conventional CEA. Before inoculation the CEA was further purified by immunoabsorbent chromatography using a highly specific anti-CEA serum. Antiserum 227 was prepared in a rabbit by giving multiple-site intradermal injections of CEA-2B distributed in the flank. The rabbit received 20 $\mu$g of CEA in 250 $\mu$l of saline emulsified with 250 $\mu$l of Freund's complete adjuvant. Similar injections were given after 40 days and serum collected every 10–14 days afterwards. Bleeds 1–6 were pooled and absorbed with freeze-dried perchloric-acid extracts of normal colon (40 mg/ml) and normal human plasma (20 mg/ml). The antiserum was checked for specificity by comparative immunodiffusion using existing antisera. The completeness of absorption was checked by immunodiffusion against normal tissue extracts and by trial radioimmunoassay performed concurrently with our existing routine assay.

*Immunodiffusion studies*

Double-diffusion reactions were carried out in 1·5% agar containing 0·9% NaCl using absorbed Antiserum 227. The various fractions were tested at the concentrations indicated in the figures.

### RESULTS AND DISCUSSION

Perchloric acid has been used extensively for the routine extraction of CEA from tumour tissues and for the pretreatment of plasma for indirect radioimmunoassay. However, it has been shown that perchloric acid can modify CEA (Rule and Goleski-Reilly, 1973*a*; Carrico and Usategui-Gomez, 1975). It is conceivable that perchloric acid could cleave sialic acid residues in CEA and modify other parts of the carbohydrate structure leading to changes in its properties.

Earlier unpublished studies from our laboratory and studies reported by Carrico and Usategui-Gomez (1975) had demonstrated that the yield of CEA obtained from colonic tumour tissue and measured by radioimmunoassay was considerably diminished by the perchloric acid treatment, and this appears to be the case with serum CEA also (Khoo, Hunt and Mackay, 1973*a*; Khoo and Mackay, 1973; Ashman *et al.*, 1977). It has been demonstrated further that CEA could be extracted from perchloric acid precipitates of tumour tissue by stirring overnight with 3M KCl solution (Rogers, 1976) or HCl at pH 3·0 (Dyce and Haverback, 1972). This indicated that some of the CEA survived irreversible denaturation by perchloric acid and was precipitated, or more likely altered in its capacity to bind to other precipitated proteins in the tissue homogenate.

*Studies on tumour tissue*

To explore the above effects more fully we have used various extraction procedures as illustrated in Fig. 1. The recoveries of CEA in the various extracts obtained from colonic, rectal and bronchial tumours are presented in Table I. About twice as much CEA can be recovered in saline extracts as from the conventional perchloric-acid extract B, thus confirming our previous observations. However, when the tissue homogenate was centrifuged and perchloric acid added to the supernatant (Extract A) the recovery of CEA was better than that in Extract B, in all but one case. In this the CEA content of the tumour was unusually high (64 $\mu$g/g). It appears from these experiments that perchloric-acid modifies some of the CEA in such a way that it binds to the insoluble part of the tissue homogenate.

The amount of CEA in perchloric acid extracts of the tumours (Table I) ranged from 0·1 to 87 $\mu$g/g tissue. The figures obtained represent radioimmunoassay values, and most of the results for Extract B are within the range quoted by Khoo *et al.* (1973*b*) for similar extracts. The higher values (64·3 and 87 $\mu$g/g) were obtained by extracting different samples from the same specimen (No. 4) of a large liver metastasis. The content of CEA in this tissue was unusually high in our experience but was typical of the CEA content of the crude extracts obtained by Coligan *et al.* (1972).

The detection of CEA in the KCl-treated perchloric-acid precipitate B-1 (Table I) has confirmed our earlier unpublished work in which the presence of CEA in the precipitates was shown by qualitative immunodiffusion reactions. The assay values were surprising, however, as they indicated in some cases that the CEA content associated with the perchloric-acid precipitate exceeded that in the corresponding extract. Similarly, CEA binding has been independently reported to occur in perchloric-acid precipitates of gastric tumours (Dyce and Haverback, 1972), and in this case no CEA was detected in the supernatant by immunodiffusion. The presence of comparatively large amounts of CEA in perchloric-acid precipitates raises questions about the use of perchloric acid for the routine preparation of CEA. The original CEA (Gold and Freedman, 1965) was extracted using phosphate-buffered saline, but more recent methods employ either perchloric-acid extraction directly on tumour homogenates (Coligan *et al.*, 1972; Krupey *et al.*, 1972) or after an initial centrifugation step (Plow and Edgington, 1975). It is therefore important to establish whether the bound CEA differs chemically from non-binding CEA or whether the effect of perchloric acid merely diminishes the yield.

Initial experiments using double diffusion have shown that CEA could also be extracted by treating the aqueous precipitate A-1 with 3M KCl solution, indicating that some molecules with CEA activity are bound to the insoluble tissue debris in the absence of perchloric acid. This has been confirmed by radioimmunoassay (Table II). Isoelectric focusing studies have shown that perchloric acid denatures or precipitates predominantly CEA molecules with isoelectric points in the range 5–9 (Rule and Goleski-Reilly, 1973). These molecules possess fewer sialic acid residues than those which focus at lower pH value (Coligan *et al.*, 1973). A low sialic acid content may therefore be a characteristic of the binding of CEA to precipitates in the presence of perchloric acid. It remains to be established if, in the absence of perchloric acid, those CEA molecules with a deficiency in sialic acid are the ones which are extracted from the precipitate. More recent work (Carrico and Usategui-Gomez, 1975) has shown that perchloric acid may also cause irreversible precipitation of CEA.

CEA could not be detected by radioimmunoassay in the soluble fractions, or KCl extracts of precipitated fractions, from several specimens of normal colon tissue. Several workers (Martin *et al.*, 1972; Pusztaszeri and Mach, 1973; Rogers *et al.*, 1974) have indicated the presence of very low amounts of CEA or CEA-like activity in normal tissues, but the amounts are often below the limit of detectability. Our results at least make it unlikely that CEA in normal colonic tissues could be masked by binding to either aqueous or perchloric-acid precipitates. These experiments also provide suitable controls showing that the assay values obtained with the tumour extracts are not due to non-specific interactions of tissue proteins in the radioimmunoassay.

*Studies on pooled sera*

The effect of perchloric acid on the quantitation of CEA in patients' sera is important in view of the different assay methods in use. In those assays where whole serum is used, the CEA values are generally higher than in indirect assays where the serum is extracted with perchloric acid (Khoo *et al.*, 1973; Ashman *et al.*, 1977). Table II compares the distribution of CEA in the perchloric-acid extract and precipitate for several pools of sera from patients with cancer. Seven out of 10 experiments showed the presence of CEA in the perchloric acid precipitate although, in contrast to tumour tissue, the amount was smaller than that in the supernatant. Our results differ from those of Sorokin, Kupchik and Zamcheck (1973) which failed to show the presence of CEA activity in serum precipitates. This discrepancy may be due to the fact that these workers used individual serum samples containing smaller amounts of CEA than our pools of sera.

The smaller amount of CEA obtained from perchloric-acid precipitates of serum than from perchloric acid precipitates of colonic tumours may be due to prior removal of CEA, probably in an asialylated form, from the circulation by the liver. If this is so, any differences in cancer specificity between perchloric-acid-soluble CEA and precipitate CEA would not be expected to show up in a comparison of the direct and indirect assays. The lower assay value obtained with the indirect assay would then more probably be due to irreversible precipitation of CEA, which takes place on treatment with perchloric acid (Carrico and Usategui-Gomez, 1975). The recent comparative study by Ashman *et al.* (1977) between a perchloric-acid extracted ammonium–sulphate precipitation assay and a whole-plasma double-antibody assay is consistent with the above concept. The study failed to show any difference in cancer discriminatory ability between the two assay systems.

*Fractionation studies on extracts from Specimen 4*

CEA in each extract obtained from Specimen 4 eluted as a single, fairly sharp

FIG. 4.—Gel filtration of extracts A and B on Sephadex G-200. Absorbance profiles A (–O–O–), B (– – –); CEA profiles A (——), B (· · ·).

band on Sepharose 6B (Fig. 2). The elution profiles on Sephadex G-200, however, were not identical. Although a major peak of CEA activity was shown for each extract, the elution volumes varied, corresponding to differences in molecular size (Fig. 3). The CEA extracted from the perchloric-acid precipitates A-2 and B-1 eluted as sharp bands corresponding to molecular sizes of 390,000 and 308,000 daltons respectively. The CEA elution and the absorbance profiles for the perchloric-acid-soluble extracts A and B show that most of the non-CEA protein in the lower mol. wt range, present in Extract A, is substantially reduced in Extract B (Fig. 4). The CEA profiles of Extracts A and B also show differences in molecular size. Extract A displays a major peak of activity with a molecular size of about 215,000 daltons and a shoulder of activity of higher molecular size (~300,000 daltons). In contrast, Extract B shows the major CEA component at an elution volume corresponding to a molecular size of 296,000 daltons, and a shoulder of CEA activity corresponding to a lower molecular size (~200,000 daltons). These differences in the elution patterns of Extracts A and B are difficult to interpret at the molecular level. They may be due to aggregation of CEA or binding of CEA to tissue proteins. Centrifuging the tumour homogenate before adding perchloric acid has the effect of removing insoluble tissue debris to which CEA would otherwise bind. The presence of CEA of large molecular size in tumour tissue has been noted previously. The CEA preparation described by Krupey *et al.* (1972) was fractionated on Sephadex G-200 into 2 peaks with CEA activity, although the bulk of the activity was present in the lower-mol. wt fraction. Coligan *et al.* (1972) on the other hand showed that the type of CEA elution profile on Sephadex G-200 depended on the tumour. Although a single peak of CEA activity with a sedimentation constant of 6·8 S was more typical, some tumours produced CEA which chromatographed with a double peak showing that a large proportion of the CEA had a higher mol. wt. The extent to which heterogeneity in molecular size in CEA is an artefact resulting from the extraction procedure is unknown, although in this context it is interesting to note that CEA with a molecular size of 370,000 daltons has been detected in both perchloric-acid-extracted serum and in whole serum from patients with cancer (Pletsch and Goldenberg, 1974).

The recovery of CEA at each stage of purification is given in Table II. In the 2 perchloric-acid extracts A and B the recoveries after G-200 chromatography were 62 and 54% respectively, typical of recoveries obtained by Coligan *et al.* (1972). The large amount of CEA lost at the Sepharose stage is difficult to account for, but this must be to some extent due to spreading of the CEA band on the column and to selection of the prominent CEA peak for further purification.

The results of concanavalin A-affinity chromatography of the partially purified extracts of CEA, obtained by Sepharose and Sephadex chromatography, are presented in Table IV. Five fractions were collected as described earlier, with most of the CEA appearing in fractions 2B, 3 and 4, and very small recoveries in the unbound peak and Peak 2A. This is in agreement with our previous studies (Rogers *et al.*, 1976) and also those of Pritchard and Todd (1976) who found the unbound material to contain predominantly non-CEA mucopolysaccharides. In the present work we have eluted tightly bound material from the column after soaking overnight in 20% methyl glucoside, as suggested by Slayter and Coligan (1976), thereby improving our total recoveries of CEA.

The recoveries of CEA in the Con A peaks of Extract B and the corresponding extract obtained from the precipitate B-1, are almost identical (Table IV). This indicates that the distribution and affinities of the Con A-binding sites are not significantly altered in CEA obtained from the perchloric-acid residue. If this were the case, quite different elution patterns for perchloric-acid-soluble and precipitated CEA would be expected. In view of this, and the known chemistry of Con A binding, together with the fact that Con A binds to the intermediate carbohydrate chains of CEA (Rogers, 1976), it may be inferred that differences in soluble CEA and CEA from perchloric-acid precipitates may be restricted to carbohydrate residues on the periphery of CEA. Our result, although providing only indirect evidence, appears to be compatible with the possible role sialic acid might play in the susceptibility of CEA to precipitation in the presence of perchloric acid. The different elution patterns on Con A obtained with Extracts A and A-1, compared to those obtained with the Extracts B and B-1 just described, may be correlated with the differences in molecular size described earlier, but they are difficult to interpret in detail. It may be concluded, however, that the differences result from an overall effect of the different extraction procedures on the chemical structure of the carbohydrate chains in CEA. It appears that, to some extent, heterogeneity of CEA, as demonstrated by Con A affinity chromatography, is dependent on chemical changes in the carbohydrate caused by the action of perchloric acid. Whether carbohydrate heterogeneity is also due to aggregation of CEA or binding to other glycoproteins is unknown.

The recoveries of CEA in the Con A fractions obtained from the aqueous extract of Specimen 4 (Table IV) show similarities with the extracts discussed above. However, by treating the Con A fraction 2B obtained from the aqueous extract, with perchloric acid, its Con A binding is altered (Table IV). After treatment and rechromatography on Con A-Sepharose almost 60% of CEA-2B was eluted in Peaks 1, 3 and 4. Although not surprising, this is an important result, since it demonstrates directly that perchloric acid alters the carbohydrate structure in CEA obtained by aqueous extraction, and suggests that most preparations of CEA in current use are chemically different from native CEA. It will be necessary to determine whether perchloric acid causes similar chemical alterations in circulating CEA.

*Immunological studies on purified extracts of CEA*

Immunological identity between CEA from the various extracts after purification has been demonstrated by immunodiffusion using Antiserum 227 (Fig. 5). Comparative binding studies, demonstrating the ability of CEA B and B-1 to inhibit the binding of labelled CEA and Antiserum 227, produced superimposable curves (Fig. 6), providing further evidence for immunological identity between perchloric-acid-soluble CEA and CEA from the perchloric-acid precipitate. Fig. 5 also shows the immunodiffusion results for CEA-2B purified by Con A affinity chromatography from various extracts, including an aqueous extract of tumour. Again a line of identity was obtained showing the presence of the conventional CEA determinant. Although more difficult to see in the stained gel (Fig. 5) original observation of the immunodiffusion plate clearly showed the presence of an additional precipitin line in the case of the purified aqueous extract, which was partly hidden and much weaker in the perchloric-acid extracts. This suggests the presence of 2 distinct antigens, the CEA antigen and a second antigen which appears to be relatively unstable, or less soluble, in perchloric acid. The presence of 2 distinct antigens in aqueous extracts of tumour has also previously been reported by Carrico and Usategui-Gomez (1975).

FIG. 5.—The upper diagram shows immunological identity on immunodiffusion between CEA extracts purified on Sephadex G-200 (outer wells) and antiserum 227 (centre well). The concentrations for extracts A, A-1, A-2, B and B-1 are 320, 96, 55, 157 and 198 μg/ml respectively. The lower diagram shows immunodiffusion patterns obtained with Con-A fraction 2B obtained from the various extracts (outer wells) and antiserum 227 (centre well). Note the double line produced by the aqueous extract. The CEA concentrations for the aqueous extract, extracts A-2, B, A-1, B-1 and A are 18, 7, 150, 18, 55 and 55 μg/ml respectively.

FIG. 6.—Comparative binding curves of CEA B (○) and B-1 (△) demonstrating the inhibition of binding of $^{125}$I-CEA (assay standard) to antiserum 227.

The second antigen may be similar to our previously described B-antigen which was also detected in purified CEA preparations after Con A fractionation, and by using conventional anti-CEA antisera (Rogers *et al.*, 1974, 1975). Our previous difficulties experienced in isolating this antigen from perchloric-acid extracts of tumour may be due to its instability or low solubility in acid, and we are now attempting the isolation of the B-antigen from aqueous extracts in order to compare it with the second antigen described above.

It is clear from the present study that perchloric acid alters the chemical structure of CEA. This manifests itself by alteration of the Con A binding sites of CEA, causing different fractionation patterns with Extracts A and B, and also with the aqueous extract before and after perchloric acid treatment. The effect of perchloric acid is also shown by modification of some forms of CEA so that they bind to insoluble tissue precipitates and are no longer soluble. It also appears that perchloric acid may affect different species of CEA in different ways. Some species appear to remain immunologically intact and soluble in perchloric acid, others are modified and bind to tissue homogenates but remain immunologically intact, whereas others again are irreversibly denatured, causing diminished overall yields of CEA.

The implications of the present study on our recently published work (Rogers *et al.*, 1977) on the disease specificity of Con A fractions of perchloric-acid-treated serum CEA, are difficult to ascertain until the chemical effects of perchloric acid on serum CEA have been established. Nevertheless our initial work suggested that heterogeneity of the carbohydrate appears to give rise to CEA molecules which vary in specificity for local and metastatic cancer, and that this specificity appeared not to be impaired by the action of perchloric acid.

*Extraction of CEA from foetal colon*

The recoveries of CEA in the crude extracts A, A-1 and A-2 of foetal colon are shown in Table V together with percentage recoveries obtained from fractions produced by Con A affinity chromatography. In contrast to colorectal tumour CEA, there appeared to be a marked increase in the CEA-like activity of the aqueous extract when this was treated with 2M perchloric acid. This suggests that perchloric acid is capable of dissociating possible complexes and exposing CEA-like activity which may be masked in the aqueous extract. Whether the increase in the assay value is due to CEA or an unrelated protein which inhibits or interferes in our CEA radioimmunoassay is unknown, and must wait further experimentation. However, non-specific interference in the assay by high salt concentration or low pH can be ruled out because of the extensive dialysis carried out on the fractions before assay.

In contrast to colorectal tumour CEA, a significant proportion of foetal CEA-like activity was not bound by Con A. The CEA which did bind in each case appeared predominantly in Fraction 2B, with very little in Fraction 3. This binding is similar to that found when sera of patients with local cancer were chromatographed on Con A-Sepharose (Rogers *et al.*, 1977). Comparison between the recoveries for the Con A fractions of aqueous and perchloric-acid-treated foetal CEA also show that part of the CEA in the unbound fraction and Fraction 3, on treatment with perchloric acid, is either denatured or irreversibly bound to the Con A-Sepharose. Undoubtedly, further work is required to substantiate these results, but the striking differences described in these preliminary experiments between colorectal CEA and foetal CEA are interesting and may suggest important differences in the structure of the carbohydrate chains.

The authors wish to thank Dr S. D. Lawler of the Department of Cytogenetics and Immunology, The Royal Marsden Hospital, London, for providing the foetal tissues. We are also grateful for the invaluable assistance of Mrs J. Dent and her staff in our Department in the collection of other tissues used in this study. Our appreciation is also extended to Professor K. D. Bagshawe and Dr M. Wass for constructive criticism and to Mrs J. Wood for assistance in preparing the antiserum. The work was aided by the Medical Research Council.

### REFERENCES

ASHMAN, L. K., LUDBROOK, J. & MARSHALL, V. R. (1977) Comparison of Whole Plasma and Perchloric Acid-extracted Plasma Assays for Carcinoembryonic Antigen. *Clin. chim. Acta*, **74**, 77.

BRATTAIN, M. G., JONES, C. M., PITTMAN, J. M. & PETLOW, T. G., II (1975) The Purification of Carcinoembryonic Antigen by Glutaraldehyde Cross-linked Concanavalin A. *Biochem. biophys Res. Comm.* **65**, 63.

CARRICO, R. J. & USATEGUI-GOMEZ, M. (1975) The Isolation of Carcinoembryonic Antigen from Tumour Tissue at Neutral pH. *Cancer Res.*, **35**, 2928.

COLIGAN, J. E., LAUTENSCHLEGER, J. T., EGAN, M. L. & TODD, C. W. (1972) Isolation and Characterization of Carcinoembryonic Antigen. *Immunochemistry*, **9**, 377.

COLIGAN, J. E., HENKART, P. A., TODD, C. W. & TERRY, W. D. (1973) Heterogeneity of the Carcinoembryonic Antigen. *Immunochemistry*, **10**, 591.

DARCY, D. A., TURBERVILLE, C. & JAMES, R. (1973) Immunological Study of Carcinoembryonic Antigen (CEA)—and a Related Glycoprotein. *Br. J. Cancer*, **28**, 147.

DYCE, B. J. & HAVERBACK, B. J. (1972) Tumour Antigen (CEA) in Gastric Adenocarcinoma Masked by a Binding Substance, likely an Antibody. In Proc. 2nd Conf. and Workshop *Embryonic and Foetal Antigens in Cancer*. Oak Ridge, USAEC Report Conf. 720208. p. 247.

GOLD, P. & FREEDMAN, S. O. (1965) Demonstration of Tumour Specific Antigen in Human Colonic Carcinomata by Immunological Tolerance and Absorption Techniques. *J. exp. Med.*, **121**, 439.

KHOO, S. K. & MACKAY, I. R. (1973) Carcinoembryonic Antigen in Serum in Diseases of the Liver and Pancreas. *J. clin. Path.*, **26**, 470.

KHOO, S. K., HUNT, P. S. & MACKAY, I. R. (1973*a*) Studies of Carcinoembryonic Antigen in Whole and Extracted Serum in Ulcerative Colitis. *Gut*, **14**, 545.

KHOO, S. K., WARNER, N. L., LIE, J. T. & MACKAY, I. R. (1973*b*) Carcinoembryonic Antigenic Activity of Tissue Extracts: A Quantitative Study of Malignant and Benign Neoplasms Cirrhotic Liver Normal Adult and Foetal Organs. *Int. J. Cancer*, **11**, 681.

KRUPEY, J., WILSON, T., FREEDMAN, S. O. & GOLD, P. (1972) The Preparation of Purified Carcinoembryonic Antigen of the Human Digestive System from Large Quantities of Tumour Tissue. *Immunochemistry*, **9**, 617.

MARTIN, F., MARTIN, M. S., BORDES, M. & BOURGEAUX, C. (1972) The Specificity of Carcinofoetal Antigen of the Human Digestive Tract Tumours. *Eur. J. Cancer*, **8**, 315.

PLETSCH, Q. & GOLDENBERG, D. M. (1974) Molecular Size of Carcinoembryonic Antigen in the Plasma of Patients with Malignant Disease. *J. natn. Cancer Inst.*, **53**, 1201.

PLOW, E. G. & EDGINGTON, T. S. (1975) Association of an Isomeric Species of Carcinoembryonic Antigen with neoplasia of the Gastrointestinal Tract. *New Engl. J. Med.*, **293**, 103.

PRITCHARD, D. G. & TODD, C. W. (1976) Purification of Carcinoembryonic Antigen by Removal of Contaminating Mucopolysaccharides. *Cancer Res.*, **36**, 4699.

PUSZTASZERI, G. & MACH, J. P. (1973) Carcinoembryonic Antigen (CEA) in Non-digestive Cancerous and Normal Tissues. *Immunochemistry*, **10**, 197.

ROGERS, G. T., SEARLE, F. & BAGSHAWE, K. D. (1974) Heterogeneity of Carcinoembryonic Antigen and its Fractionation by Con A Affinity Chromatography. *Nature, Lond.*, **251**, 519.

ROGERS, G. T., SEARLE, F. & WASS, M. (1975) Immunological and Chemical Studies on Subfractions of Carcinoembryonic Antigen. *Immunochemistry*, **12**, 839.

ROGERS, G. T. (1976) Heterogeneity of Carcinoembryonic Antigen. Implication on its Role as a Tumour Marker Substance. *Biochem. biophys Acta*, **458**, 355.

ROGERS, G. T., SEARLE, F. & BAGSHAWE, K. D. (1976) Carcinoembryonic Antigen: Isolation of a Sub-fraction with High Specific Activity. *Br. J. Cancer*, **33**, 357.

ROGERS, G. T., LEAKE, B. A., SEARLE, F. & BAGSHAWE, K. D. (1977) Heterogeneity and Specificity of Circulating Carcinoembryonic Antigen. *Eur. J. Cancer*, **13**, 293.

RULE, A. H. & GOLESKI-REILLY, C. (1973*a*) Carcinoembryonic Antigen (CEA): Separation of CEA-reacting Molecules from Tumour, Foetal Gut, Meconium and Normal Colon. *Immunol. Comm.*, **2**, 213.

RULE, A. H. & GOLESKI-REILLY, C. (1973*b*) Carcinoembryonic Antigen (CEA) "Fingerprints". *Br. J. Cancer*, **28**, 464.

SLAYTER, H. S. & COLIGAN, J. E. (1976) Characterization of Carcinoembryonic Antigen Fractionated by Concanavalin A Chromatography. *Cancer Res.* **36**, 1696.

SOROKIN, J. J., KUPCHIK, H. Z. & ZAMCHECK, N. (1973) Carcinoembryonic Antigen in Colon Cancer: Absence in Perchloric Acid Precipitates of Plasma. *J. natn. Cancer Inst.*, **51**, 1081.

WHITAKER, J. R. (1963) Determination of Molecular Weights of Proteins by Gel Filtration on Sephadex. *Analyt. Chem.*, **35**, 1950.